# Usefulness of convalescent plasma transfusion for the treatment of severely ill COVID-19 patients in Pakistan

**DOI:** 10.1186/s12879-021-06451-7

**Published:** 2021-09-27

**Authors:** Tehmina Nafees Sonia Khan, Samina Naz Mukry, Shahtaj Masood, Lubna Meraj, Bikha Ram Devrajani, Javed Akram, Naveena Fatima, Sidra Maqsood, Ayesha Mahesar, Roomana Siddiqui, Sadia Ishaque, Muhammad Bilal Afzal, Sanam Mukhtar, Sara Ahmed, Arshi Naz, Tahir Sultan Shamsi

**Affiliations:** 1 National Institute of Blood Diseases & Bone Marrow Transplantation, Karachi, Pakistan; 2 Hayatabad Medical Complex, Peshawar, Pakistan; 3 Rawalpindi Medical University, Rawalpindi, Pakistan; 4 Liaquat University of Medical and Health Sciences, Jamshoro, Pakistan; 5 University of Health Sciences, Lahore, Pakistan; 6 The Indus Hospital, Karachi, Pakistan; 7 Regional Blood Center, Sukkur, Pakistan; 8 Orthopedic and Medical Institute (OMI) Hospital, Karachi, Pakistan; 9 Liaquat National Hospital, Karachi, Pakistan; 10 Shah Medical Center, Karachi, Pakistan; 11 Saifee Hospital, Karachi, Pakistan; 12 Patel Hospital, Karachi, Pakistan

**Keywords:** Convalescent CP recipient, Convalescent plasma (CP), COVID-19, SARS CoV2 infection, SOFA score

## Abstract

**Background:**

Convalescent plasma(CP) was utilized as potential therapy during COVID-19 pandemic in Pakistan. The study aimed at appraisal of CP transfusion safety and usefulness in COVID pneumonia.

**Methods:**

Single arm, MEURI study design of non-randomized open label trial was conducted in five centers. Patients werecategorized as moderately severe, severe, and critical. The primary endpoint was a) improvement in clinical status and change in category of disease severity; secondary endpoint was b) CP ability to halt disease progression to invasive ventilation. CP transfused to hospitalized patients. Statistical tests including median (interquartile ranges), Mann-Whitney U test, Fisher’s exact test using SPSS ver. 23, ANOVA and Chi-square test were applied for the analysis of results parameters before and after CP treatment. SOFA score was applied for multiorgan failure in severe and critical cases.

**Results:**

A total of 50 adult patients; median age 58.5 years (range: 29–92 years) received CP with infusion titers; median 1:320 U/mL (Interquartile range 1:80–1:320) between April 4 to May 5, 2020. The median time from onset of symptoms to enrollment in trial was 3 to 7 days with shortness of breath and lung infiltration as severity criterion. In 35 (70%) recipients, oxygen saturation improved from 80 to 95% within 72h, with resolution of lung infiltrates. Primary endpoint was achieved in 44 (88%) recipients whereas secondary endpoint was achieved in 42 (84%). No patient experienced severe adverse events. A high SOFA score (> 7) correlated with deaths in severe and critical patients. Eight (16%) patients expired due to comorbidities; cardiac arrest in 2 (4%), multiorgan failure secondary to cytokine storm in 5 (10%) and ventilator associated complications in 1 (2%).

**Conclusion:**

CP transfusion can be used as a safe and useful treatment in moderately severe and severe patients.

**Trial registration:**

The trial registration number is NCT04352751 (https://www.irct.ir/search/result?query=IRCT20200414047072N1). Trial Registration date is 28th April 2020.

## Introduction

Severe acute respiratory syndrome corona virus 2 (SARS-CoV-2) is a novel member of Coronaviridae family [1]. It is a positive sense RNA beta-corona virus possibly evolved as a result of animal spill over from bats [2]. The infection surfaced in late 2019 and took the form of a pandemic. To-date the virus has infected 20 million people in 215 countries or regions resulting in 7.6 lacs deaths (https://www.worldometers.info/coronavirus/> [Accessed 14 August 2020]. Although the mortality rate due to novel coronavirus disease (COVID-19) is slightly low (17.5%) compared to SARS (severe acute respiratory syndrome) and MERS (Middle East Respiratory Syndrome), the level of transmission is high which has resulted in recent pandemic of unprecedented consequences. No prophylactic or curative treatment options exist for COVID-19 at present [3]. As per recent trials, antivirals against HIV and other coronaviruses such as protease inhibitor combination lopinavir/ritonavir have failed to prove efficacy in the treatment of severe COVID-19 [4]. Although new antivirals like remdesivir is a promising candidate among other explored treatment options but the data on its safety and efficacy is still limited. Immune therapies such as the use of convalescent plasma (CP) for the treatment of infectious diseases SARS, MERS, Ebola, Measles, polio, H1N1 influenza etc., has been proven effective for more than a century especially during emergency situations [5]. Under the current condition of medical emergency CP treatment has been listed as a possible investigational treatment option for severe and critical COVID-19 cases [6].

The mechanism of action of CP treatment is diverse involving the viral neutralization and clearance by direct inactivation and/or by enhancing humoral immune response to viral antigen. Complete viral clearance generally happens 12 to 20 days post- infection [7]. Therefore, the efficacy of CP therapy could be enhanced by its administration soon after appearance of usual disease symptoms [8]. The antagonistic and synergistic effect of steroid or other experimental drug therapies may alter the efficacy of CP therapy [9].

CP therapy has been strongly advocated by many researchers as a potential therapeutic option [10]. Elevated inflammatory markers including IL-6, ferritin, C-reactive protein and Lactate Dehydrogenase (LDH) as markers of tissue injury were proved to be good predictors of viral induced cytokinemia and compromised respiratory function as a result lung parenchymal damage in COVID-19 disease [11, 12]. Interestingly, higher concentrations of IgG appeared earlier in severe COVID-19 female patients [13] but presence of anti-HLA/HPA/HNA antibodies in multiparous women discourage CP donation from female survivors to avoid the possible risk of transfusion related acute lung injury (TRALI) in recipients [14]. Although the duration of persistence of anti-SARS-CoV-2antibodies in recovered patient plasma remains unknown but based on previous studies on MERS, SARS, and Ebola it suggested that CP could be collected for up to 6 months at an interval of every 14 days [13]. Asymptomatic COVID-19 patients recovered without hospitalization in general population may also be the suitable donors. The possible adverse events such as allergy to plasma components, coinfection, multiple organ failure, risk of thrombosis and volume overload should always be taken into account while prescribing CP therapy [15].

The purpose of this study was to explore feasibility of CP as an emergency investigational new treatment option in COVID-19 patients with severe to critical disease treated in different hospitals all over Pakistan.

## Materials and methods

This MEURI (Monitored Emergency Use of Unregistered and Investigational Interventions), Non-randomized open label trial was approved by the institutional review board (NIBD/RD-02/03–2020), National Bioethics Committee of Pakistan (NBC Registration No: NBC-472 COVID19–03), Drug Regulatory Authority of Pakistan (Registration No: F.NO.17–8/2020 DD (PS) and clinicatrials.gov (NIH ID: NCT04352751). It was conducted in accordance with Declaration of Helsinki 2000. In the initial period, hospitalized patients of covid-19 from April 4, 2020 to May 5, 2020 in five different hospitals across Pakistan were included. All recipients were above 18 years, and a written informed consent was obtained from each patient or a family member. The primary and secondary endpoint of this study were, a) improvement in clinical signs and symptoms and change in category of disease severity, b) ability of CP to halt disease progression leading to invasive ventilation, respectively. CP screening for Hepatitis B virus (HBV), Human Immunodeficiency Virus (HIV) was done by chemiluminescence assay using Abbot Architect while Hepatitis C virus (HCV) was screen by HCV nucleic acid testing (NAT) using artus® RG RT-PCR kit., using complete blood counts (CBC) samples of CP donors’ and Rapid diagnostic test (RDT) and microscopy were used to screen malaria.

### Data collection

**Fig. 1:**
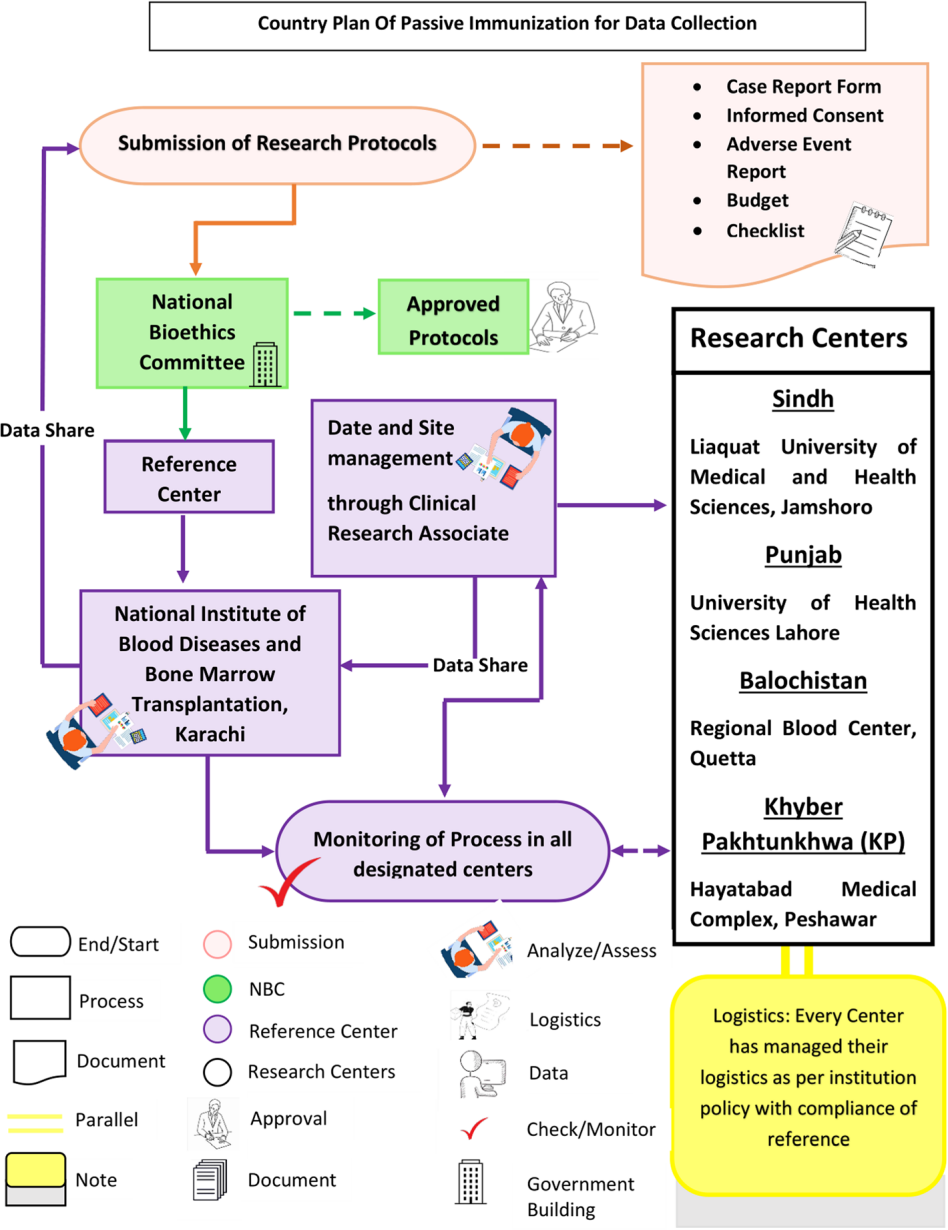
The country plan for Passive immunization explains the entire process from submission of protocol for approval to site management, data sharing and monitoring of entire trial process in all authorized centers of transfusion across Pakistan

Data was collected from the collaborative centers across Pakistan by clinical research associates through clinical report form (Fig. 1).

### Patient selection and intervention

The Polymerase chain reaction (PCR) positive COVID-19 patients were categorized in three groups according to the guidelines of center for disease control and prevention (CDC) (cdc.gov). The moderately severe category was defined as i) shortness of breath, ii) respiratory rate > 30 breaths/minute, oxygen saturation ≤ 93% on room air along with lung infiltration of 20 to 40% within 24 to 48 h of hospital admission. Severe category included patients with respiratory rate ≥ 30 breaths/minute, oxygen saturation ≤ 93% on oxygen support and lung infiltrates ≥50% within 24 to 48 h of hospital admission. The last amongst three categories i.e. critical patients were in respiratory failure, shock and multiorgan failure [16, 17].

### Inclusion and exclusion criteria

All PCR positive, hospitalized COVID-19 patients ≥18 years of age, in all three COVID-19 disease categories were included in this study. The patients with history of allergic reaction to sodium citrate or with multi-organ failure secondary to cytokine storm on ventilator for more than two days and in sepsis were excluded. Patients who received tocilizumab / remdesivir also not included in this study. All admitted patients received supportive care, while some patients received antibiotic treatment, glucocorticoid, and oxygen support as per institutional treatment protocols.

### Specimen collection

Blood samples were drawn before CP transfusion for CBC, biochemistry parameters including LDH, Serum Ferritin, and coagulation test of D-Dimer. An ABO compatible CP was transfused in a single dose of 400 mL with approximated high IgG titers (median 1:320 U/mL) and low IgG titers (< 1:160 U/mL) was transfused to patients in 5–6 h duration. World health organization (WHO) guidelines for the selection of a healthy blood donor was followed for convalescent plasma donors. The entire transfusion process was performed under supervision of clinicians at the respective hospitals with close monitoring for any possible adverse events during procedure. The vitals of every recipients before, during and after CP transfusion were recorded carefully. Both clinical and laboratory parameters were documented before and after CP transfusion to rule out any significant finding.

For the assessment of main outcome, vitals, status of saturation of oxygen on non-invasive and invasive mechanical ventilation, blood samples for laboratory investigations were obtained in 9 days post CP transfusion follow-ups.

A 6-points ordinal Sequential Organ Failure Assessment (SOFA) score was done for the evaluation of multiorgan failure and patients’ outcome. (https://clincalc.com/IcuMortality/SOFA.aspx).

### Statistical analysis

Median (interquartile ranges) were used to described continuous variables while comparison was done by Mann-Whitney U test; categorical variables were compared by Fisher’s exact test between patient groups using SPSS ver. 23. ANOVA and Chi-square test were used to compare laboratory parameters before and after CP treatment.

## Results

### General trait of participants

A total of 50 COVID-19 PCR positive hospitalized patients were selected initially with male predominance i.e., 39(78%) with a median age of 58.5 years (range: 29–92 years). Of 50 patients, 15 (30%) were moderately-severe, 33 (66%) were severe while 2 (4%) were critical. The commonest symptoms observed at the onset of disease was shortness of breath (98%) body aches (12%) and fever (8%). All patients were on broad spectrum antibiotics due to suspected bacterial infections as per institutional policy. CP was transfused in all patients with variable titers; Median 1:320, including high titers (1:160-1:320 U/mL) CP infusion done in 31 non- intubated subjects (62%) of severe group and 2(4%) in intubated critical. The low titer (1:80–1:160 U/mL) CP was transfused in non-intubated category of moderately severe 15(30%) and severe 2(4%). Total 27 recipients of high titer plasma out of 31 showed early betterment in disease status in severe category, with 18 out of 27 patients showing response in first 72 h of infusion. The remaining 9 recipients were elderly patients in severe group transfused later (after 3-7 days) and they had  delayed clinical improvement. The remaining four recipient who initially manifested good progress; expired later due to cardiac disease  and organ failures secondary to comobidities in severe  group. One patient in critical group also survived. An overall survival rate of >75% was achieved in recipients of high titer CP infusion within severe disease group. Altogether, 17 recipients of low titer CP i.e., 15 in moderately severe group and and two patients with severe COVID-19 disease  manifested very promising results in recipient’s recovery. No moderate to severe adverse reactions were observed in any of the recipients (Table 1).

**Table 1:** Demographic and clinical information of COVID-19 patients with and without underlying medical conditions

Demographics	Moderate (N + 15)	Severe (***N*** = 33)	Critical (***N*** = 2)	***p*** - value
Age (years)	63.06 ± 15.1	54.48 ± 13.2	66.0 ± 2.82	0.101^a^
Sex				
Male	11	26	2	0.682 ^b^
Female	04	07	0	
Duration of admission (days)	8.8 ± 5.4	11.8 ± 7.0	18.5 ± 12.0	0.116^a^
Smoking status	1	1	1	0.025^*b^
Co-morbids	8	23	2	0.316^b^
Ventilator	0	2	2	0.000^*b^
**Presenting complains**				
Fever	1	2	1	0.082^b^
SOB	15	33	1	0.000^*b^
Cough	0	3	0	0.440^b^
Bodyaches	3	2	1	0.093^b^
Respiratory rate ≥ 30/min	6	27	1	0.014^*b^
Arterial blood oxygen saturation ≤ 93%	10	27	1	0.355^b^
Lung infiltrates > 50% within 24 to 48	5	20	1	0.215^b^

^a^One-way ANOVA

^b^Chi-square test

*Significant value

### Clinical and laboratory parameters in CP recipients

Significantly elevated levels of ferritin, C-reactive protein and D-dimer were found in all three disease categories of COVID-19 patients (Table 2). Low hemoglobin was noted only in critical patients (9.4 ± 3.9 g/dL). High WBC counts in 25 (50%) along with raised ANC (absolute neutrophil counts) and lymphopenia in 20 (40%) before CP therapy. However, the average platelets were within normal range both pre-and post CP therapy. None of the recipients had malaria, HIV, HBV, HCV infection or any sexually transmitted disease.

**Table 2:** Changes in Lab findings and efficacy of CP in comparison to other experimental regimes

Clinical Lab Parameters	COVID-19 Disease Categories								
	**Moderate**	**Severe**	**Critical**						
**Pre CP**	**CI**	***p*-value**	**Pre CP**	**CI**	***p*-value**	**Pre CP**	**CI**	***p*-value**	
**Post CP**	**Post CP**	**Post CP**							
**Hematological Parameters (CBC)**									
Hemoglobin g/dl	13.4 ± 1.4	−0.2-1.76	0.112^c^	12.9 ± 1.5	−0.45-0.88	0.504^c^	12.4 ± 0.35	−35.7-41.0	5.00^c^
12.7 ± 1.4	12.7 ± 1.5	9.4 ± 3.9							
Total Leucocyte count × 10^9^ (TLC)	13.6 ± 6.1	−2.3-5.2	0.425^c^	13.2 ± 6.3	−1.69-3.09	0.556^c^	6.9 ± 1.8	−19.7-16.5	0.458^c^
12.1 ± 4.0	12.5 ± 6.2	8.9 ± 1.9							
Absolute Neutrophil Count (ANC)	10.5 ± 6.6	−3.5-5.8	0.600^c^	12.7 ± 14.7	−1.16-9.21	0.124^c^	45.7 ± 58.3	− 508.2-588.0	0.525^c^
9.4 ± 4.4	8.7 ± 5.0	5.85 ± 2.6							
Absolute Lymphocyte Count (ALC)	1.5 ± 1.3	−1.94- -0.4	0.006^*c^	1.60 ± 1.58	−2.06- -0.29	0.011^*c^	4.5 ± 4.9	−18.4-23.3	0.376^c^
2.7 ± 1.1	2.78 ± 1.91	2.0 ± 2.5							
Platelets Count × 10^9^	227.5 ± 6.3	− 997.9-818.9	0.429^c^	341 ± 99	−50.0-32.5	0.637^c^	286.5 ± 164.7	− 585.1-876.1	0.240^c^
317 ± 94.7	350 ± 9	141.0 ± 83.4							
**Biochemistry Markers**									
Alanine aminotransferase (ALT) U/L	91.0 ± 114	−54.7-186.3	0.220^c^	30.1 ± 21.9	−14.8-16.8	0.892^c^	135 ± 1.4	− 155.7-876.1	0.79^c^
25.1 ± 14.3	29.2 ± 5.6	74.5 ± 12.0							
Aspartate Aminotransferase (AST) U/L	97.0 ± 62.0	− 77.3-229.3	0.167^c^	44.5 ± 23.5	−1.97-17.9	0.084^c^	128 ± 1.41	− 132.6-70.6	0.161^c^
21.0 ± 14.9	36.5 ± 18	97.0 ± 9.8							
Bilirubin mg/dl	0.32 ± 0.95	−0.24- 0.29	0.789^c^	2.1 ± 5.2	−1.69-4.20	0.362^c^	0.54 ± 0.21	−0.65-0.74	0.563^c^
0.30 ± 0.24	0.85 ± 1.11	0.50 ± 0.14							
Creatinine mg/dl	1.1 ± 0.56	−0.59- 0.59	1.000^c^	1.07 ± 1.05	−0.17-0.33	0.508^c^	1.75 ± 1.2	−12.5-10.9	0.536^c^
1.1 ± 0.59	0.99 ± 1.3	2.5 ± 0.1							
LDH U/L	404 ± 244	224.1–236.8	0.001^*c^	450.67 ± 192	− 242.0-822.0	0.144^c^	250 ± 70.7	− 592-563	0.804^c^
173 ± 65	160 ± 27	235 ± 6.3							
**Inflammatory Markers**									
C-Reactive Protein (CRP) mg/L	28.1 ± 34.1	2.69–43.2	0.030^*c^	53.0 ± 91.2	8.17–65.17	0.014^*c^	159.3 ± 204	− 2813-2778	0.949^c^
5.1 ± 2.4	16.4 ± 1.3	141.7 ± 107.1							
Ferritin ng/ml	1000 ± 929	50.8–1404.5	0.038^*c^	1583 ± 1267	− 311.0-1034.2	0.011^*c^	768.5 ± 144.9	− 1026-2213	0.135^c^
272 ± 126	558 ± 93	175.0 ± 35.3							
D-Dimer ng/ml	1.5 ± 0.45	−2.20-2.07	0.906 ^c^	399.9 ± 460	275.3–1772.9	0.186^c^	78.0 ± 28.2	− 173.3-309.5	0.173^c^
1.6 ± 0.64	38.2 ± 7.4	9.9 ± 1.4							
**Treatment**									
Antibiotics	3	0.064^d^	29	0.000^*d^	2	0.500^d^			
8	13	1							
Corticosteroid	14	0.165^d^	27	0.195^d^	2	0.500^d^			
11	23	1							

^c^Paired sample t-test

^d^Fisher’s Exact test

^*^Significant value

### Post CP transfusion response (72 hours)

**Fig. 2:**
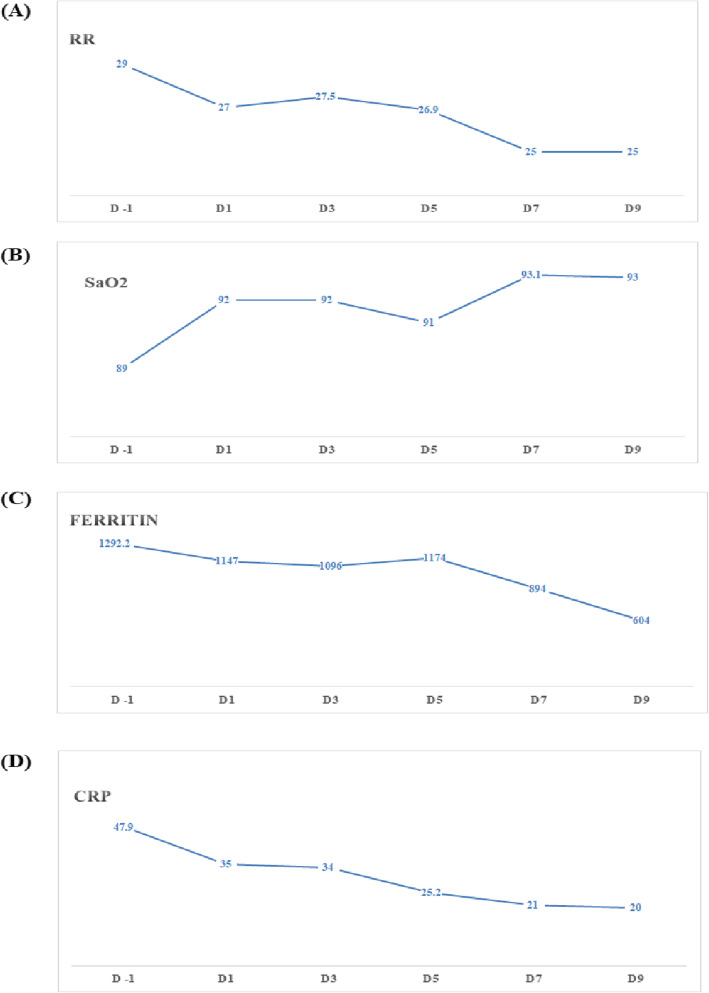
Manifests the initial response as decline in raised values of Respiratory rate, CRP, ferritin, and increased Oxygen saturation after CP administration in first 72 h

A marked improvement in SaO_2_ level i.e., from 80% of SaO_2_ to 95% was recorded in CP recipients. The respiratory rate of ≥30 breaths/minutes in 31(62%) out of 50, strikingly subsided to normal during first 3 days. A marked decline was also observed in markedly raised pre-CP inflammatory markers specifically ferritin;(Pre CP: Median 350 (409–1415) in moderately severe group (Post-CP: Median 250;(IQR 220–353) (*p* < 0.038) and in severe patients (Pre-CP: Median 1091 (666–2254) (Post-CP; 241 (150–427) and (*p* < 0.011). Also C-reactive protein (*p* < 0.030) and (*p* < 0.014) in first two disease categories respectively showed decrease along with D-dimer values in severe and critical patients, over the period of 72 h (Fig. 2).

**Fig. 3:**
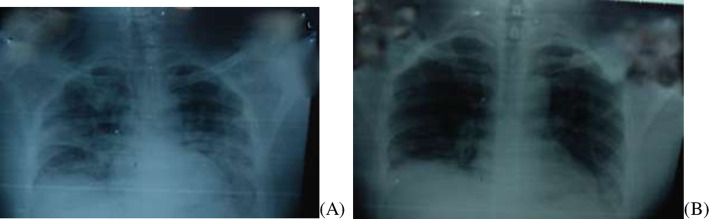
Radiological findings on chest X-ray of COVID-19 positive patient. **A** Pre-CP findings exhibits patchy infiltrations bilaterally. **B** Post CP CXR shows absorption of pulmonary infiltrates and improvement bilaterally on 5th day of CP transfusion

Initial radiological findings supported symptoms of acute respiratory distress syndrome (ARDS) with bilateral presentation of opacities in most patients affecting the middle and lower zones. It was interesting to note that apparent absorption of interstitial pneumonia was observed as early as 4 days post CP in most patients (Fig. 3).

Significantly improved LDH values (*p* = 0.043) also confirmed hepatic improvements among CP recipients. Aspartate aminotransferase (AST) also improved with CP treatment. A gradual decline seen in WBC, ANC with further improved lymphocytes counts over the period of 5-9 days post CP transfusion follow-up (Table 2).

### Correlation between comorbidities and CP transfusion

Underlying co-morbidities were observed in 33 (66%) CP recipients in all three categories including asthma in 1 (2%), malignancy in 1 (2%), diabetes in 5 (10%), hypertension in 10 (20%), diabetes + hypertension in 13 (26%) and 03(6%) had hypertension +ischemic heart disease (IHD). Three male recipients with severe COVID-19 had a history of smoking (Table 1). Four out of 5 diabetics got better and discharged after CP therapy; one patient died due to comorbidity and ventilator related complication in severe disease category. Nine (18%) patients had hypertension and got discharged after achieving primary and secondary endpoints, two patients with hypertension and ischemic heart disease also expired in severe disease group. 11 (22%) out of 13 patients of diabetes + hypertension improved clinically, and 2 (4%) patients died because of associated heart disease and renal failure in severe disease group. One patient with malignancy; later shifted on ventilator also expired in severe group whereas asthmatic patient significantly improved after CP infusion. One patient in moderately severe group expired due to sudden cardiac arrest in the absence of any known preexisting comorbid.

### SOFA score

The 6-point ordinal SOFA scoring of all patients was calculated and it was observed that six recipients who expired later had a high score > 6 other than those two patients who died of cardiac and malignancy issues and had sofa score < 6. These six patients were shown to have high percentage of estimated mortality. One recipient out of six subjects was already on ventilator. The remaining five patients expired due to cardiac arrest and multi organ failure secondary to cytokine syndrome and other associated comorbid. The calculated score in recipient on ventilator and in multi organ failure was 7–10 points and in non-vent recipients it was 4–7 points.

### Primary and secondary end points achieved

Out of 50 recipients, 42 (84%) patients, achieved both primary and secondary outcomes of disease severity reversal, clinical improvement and subsided laboratory parameters to baseline values thereby preventing disease progression. The remaining two patients who achieved primary endpoint initially (4%) expired due to sudden cardiac arrest and malignancy. Two subsequent negative PCR were obtained within 9 days for all discharged CP recipient. The overall duration of admission was shorter in patients with no underlying medical condition i.e., 3–18 days vs 3–25 days (for patients with existing co-morbid). The 35(70%) patients who received CP within 7 days of a positive COVID-19 RT-PCR (or onset of their illness) recovered within 13 days (median = 9 days) as compared to those who received it late.

**Fig. 4:**
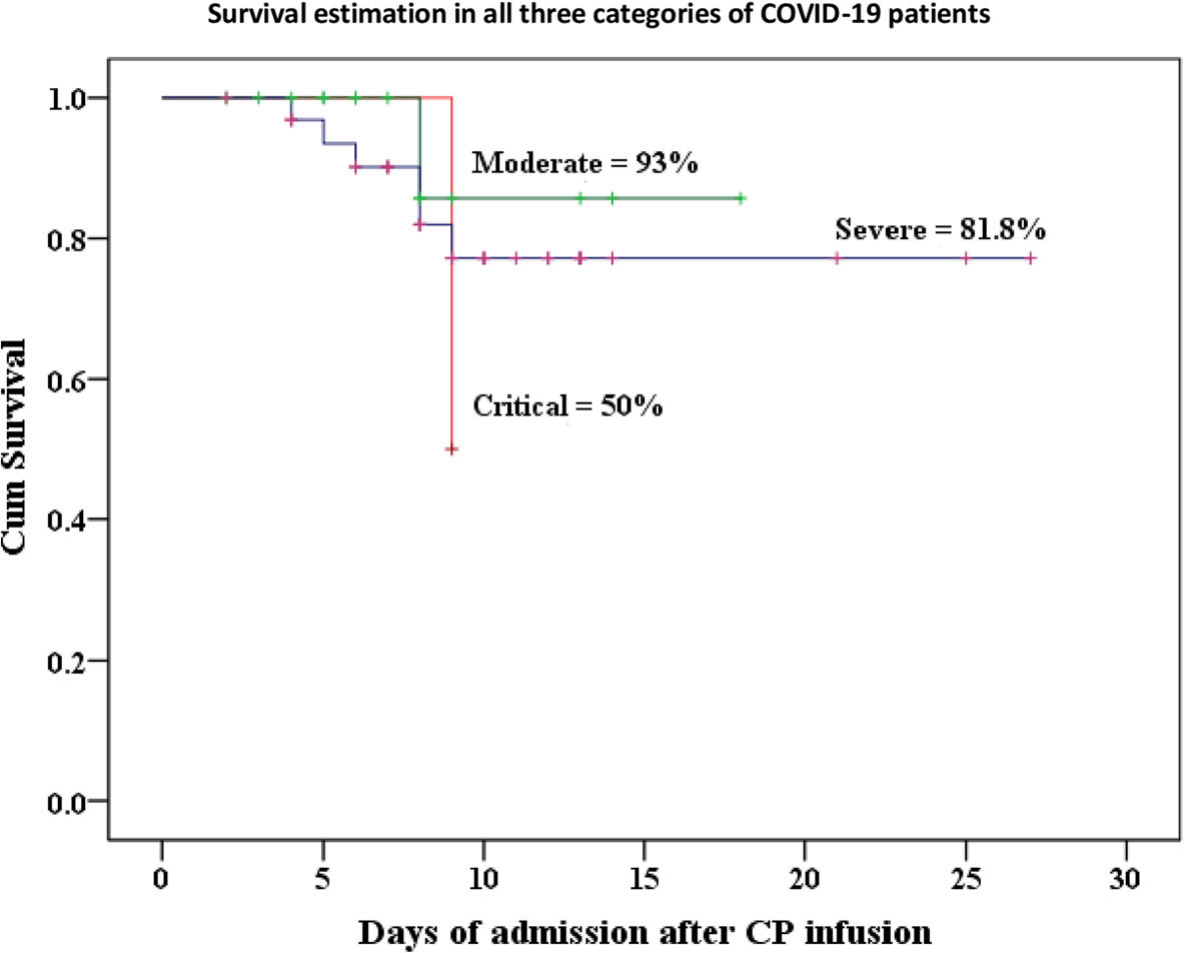
Survival curve of moderate, severe, and critical COVID-19 CP recipient patients through Kaplan-Meier analysis

Interestingly, one patient was successfully extubated from ventilator on fifth day of post CP and manifested marked improvement in oxygen saturation and chest x-ray findings. The other patient on ventilator receiving CP treatment died of ventilator related complication including excessive cytokine storming as confirmed by deranged inflammatory markers (Fig. 4).

## Discussion

This MEURI designed study was conducted for the assessment of CP’s safety and usefulness for COVID-19 hospitalized patients in Pakistan and achievement of primary and secondary end points i.e., ‘improvement in symptoms as well as change in clinical severity of disease’ and ‘halting disease progression’. The complete viral clearing was confirmed by negative RT-PCR**.** Both primary and secondary endpoints were achieved in moderately severe (100%) and severe COVID-19 patients (82.35%) with no severe adverse events. The oxygen saturation was markedly improved within 3 days after receiving a single dose of CP transfusion with improved pulmonary lesions. The SARS CoV2 causes pneumonia possibly due to its rapid replication leading to massive infiltration of neutrophils and lymphocytes. The accumulation of pro-inflammatory cytokines in alveoli may cause cytokine storm leading to acute lung injury and acute respiratory distress syndrome (ARDS) in most patients [2]. COVID-19 is characterized by tissue injury as a result of alveolar capillary micro thrombi more prevalent in COVID patients leading to tissue hypoxia [18] and in our dataset raised levels of non-specific tissue injury marker i.e. LDH was observed in patients before CP treatment which sharply reduced after CP transfusion (Table 2 [10]). The overall steady improvement in lymphocyte counts, ferritin, D-Dimer, pulmonary lesions and ultimately marked betterment in oxygen saturation along with clear chest X-ray possibly occurred due to neutralizing antibodies in CP that lead to cessation of replication and eradication of damaging virus from the blood stream as well as lung parenchyma [13]. This possible mechanism also aided in clearance of virus resulting in negative PCR in 80% of patients on fifth day of transfusion of CP. This rapid action of CP against COVID − 19 shows that it is indeed a promising candidate for treatment of COVID-19 with rare side effects. The hemoglobin in our results remained in norms except in critical group where it was 9.8 g/dL. The suggested mechanism for low Hb in critical COVID patients may be due to viral inhibition of heme metabolism or virus-induced hemoglobin denaturation [19].

A recent clinical trial observed sustained or improved supplemental oxygen requirements (odds ratio: 0.86; *p* = 0.028) which is in-line with present study [20]. A considerably important factor is the time of CP infusion. In a previous study on SARS, better treatment outcome was achieved on CP infusion before 14th day of infection. Similarly, in the present study, CP was transfused between 3 and 7 days of infection. SARS CoV2 infections has been reported to be fatal in elderly age group with co-morbidities such as hypertension, diabetes, asthma [17]. In the present study, the patients who could not survive even after timely CP treatment were either elderly or with underlying medical conditions or both. Although one patient had asthma, but he recovered due to timely CP treatment [21]. Asthma was initially thought to be a risk factor for fatal outcome in COVID-19 but it has been reported to have protective effect in recent literature [22]. SOFA scoring applied to the recipients who expired post CP transfusion, supported the fact that patients on ventilator and those who received delayed CP transfusion after a week or more of hospitalization had poor prognosis as compared to those who were not on ventilator and received CP therapy much earlier within 7 days of hospitalization. Hence it can be stated that prolonged stay and delayed CP therapy might lead to the development of multi organ damage leading to complications specifically in patients on ventilator and ultimately resulted in increased number of deaths. Transfusion related acute lung injury has been reported in the past in a female CP recipient for treatment of Ebola [13]. In the present study, no such or other adverse events were recorded for any of the CP treated patients. Recent CP trials on COVID-19 patients from China and USA also reported no side effects of CP transfusion.

Dose of CP for efficient treatment is an important factor [12, 14]. Suboptimal dose CP may have rare possibility of suppression of recipient’s innate immune response against virus resulting in antibody induced enhancement of infection. The CP donor screening for SARS CoV2 via PCR is recommended to exclude the possibility of viral transmission to recipient possibly increasing viral load in already infected patients. During the present study, this precautionary screening was performed for every donor.

Hence, the CP transfusion so far, seemed to be the safest modality of managing COVID-19 patients and the hall mark of this therapy is timely infusion and selection of category of recipients. No promising results were obtained in patients with delayed infusion and critical group on ventilator along with concomitant comorbid. Limitation of this study is that there was no randomized controlled group here, so no inference on efficacy of CP can be drawn. Randomized controlled trials are needed to study the benefits or disadvantages  of CP transfusion in COVID-19 patients.

## Conclusion

Based on the current findings it can be concluded that the CP can be considered as safe, low cost and handy investigational treatment option with very uncommon side effects during the present state of crisis for management of moderate to severe COVID-19 patients. No severe adverse events were observed in patients receiving CP treatment. However, the time of initiation of CP treatment is critical. The sooner it is deployed, the better and quicker would be the desired outcomes.

## Data Availability

The data that support the findings of this study are available from the corresponding author, upon request.

## Abbreviations

CP: Convalescent Plasma
PCR: Polymerase Chain Reaction
TRALI: Transfusion Related Acute Lung Injury
ANC: Absolute Neutrophil Counts
LDH: Lactate Dehydrogenase
SOFA: Sequential Organ Failure Assessment
